# Multimodal ultrasound and computed tomography imaging may improve the discrimination between symptomatic and asymptomatic patients with carotid stenosis

**DOI:** 10.1016/j.ahjo.2026.100898

**Published:** 2026-09-16

**Authors:** Simon Stemmler, Martin Soschynski, Martin Czerny, Matthias Siepe, Thomas Zeller, Dirk Westermann, Roland-Richard Macharzina

**Affiliations:** a Department of Cardiology and Angiology, University Heart Center Freiburg - Bad Krozingen, Faculty of Medicine, University of Freiburg, Germany; b Department of Anesthesiology, University Medical Center Hamburg-Eppendorf, Germany; c Department of Diagnostic and Interventional Radiology, University Medical Center Freiburg, Faculty of Medicine, University of Freiburg, Germany; d Department of Cardiovascular Surgery, University Heart Center Freiburg – Bad Krozingen, Germany; e Department of Cardiac Surgery, Inselspital, Bern University Hospital, University of Bern, Bern, Switzerland

**Keywords:** Carotid stenosis, Plaque imaging, Ultrasound, Computed tomography, Stroke

## Abstract

**Study objective:**

To evaluate whether ultrasound (US) plaque features are associated with plaques from symptomatic patients and whether the combination with computed tomography (CT) parameters improves diagnostic accuracy.

**Design:**

Single-center prospective cohort study.

**Setting:**

Tertiary academic center for cardiovascular medicine in Germany.

**Participants:**

188 patients who underwent carotid endarterectomy (CEA) with 156 receiving US and 152 receiving CT.

**Interventions:**

US assessment of plaque echogenicity, heterogeneity, calcification, discrete white areas, juxtaluminal echolucency. CT assessment of calcified plaque volume and heterogeneity. Multimodal combination of US and CT parameters.

**Main outcome measures:**

Comparison of plaque features between patients with symptomatic and asymptomatic carotid stenosis. Diagnostic performance using logistic regression, receiver operating characteristic (ROC) curve analysis and area under the curve (AUC).

**Results:**

Patients with symptomatic carotid stenosis had lower echogenicity (plaque with gray-scale (GS) <25: 61.6 ± 35.9% vs. 30.9 ± 27.4%, *P* < 0.001), greater US heterogeneity (coefficient of variation (CV): 1.20 ± 0.54 vs. 0.82 ± 0.48, *P* < 0.001), and more juxtaluminal echolucency (23.2% vs. 3.0%, *P* < 0.001). Plaque with GS <25 achieved an AUC of 0.76 (95% confidence interval, 0.68–0.84). Combining this US measure with CT-derived heterogeneity yielded a higher AUC of 0.83 (0.74–0.92), *P* = 0.062, with 80.0% sensitivity and 82.0% specificity.

**Conclusions:**

US identifies patients with symptomatic carotid stenosis via echogenicity, heterogeneity, and qualitative features. Combining US with CT may improve diagnostic accuracy and warrants further validation. Multimodal plaque imaging should be evaluated concerning prospective stroke risk to assess its role in risk stratification before CEA.

## Highlights

• Ultrasound plaque features accurately distinguished symptomatic from asymptomatic patients with an AUC of 0.76.
• Combining ultrasound echolucency with CT heterogeneity achieved an AUC of 0.83.
• Simple multimodal criteria may support carotid plaque risk stratification in future research.

## Graphical abstract

**Figure:**
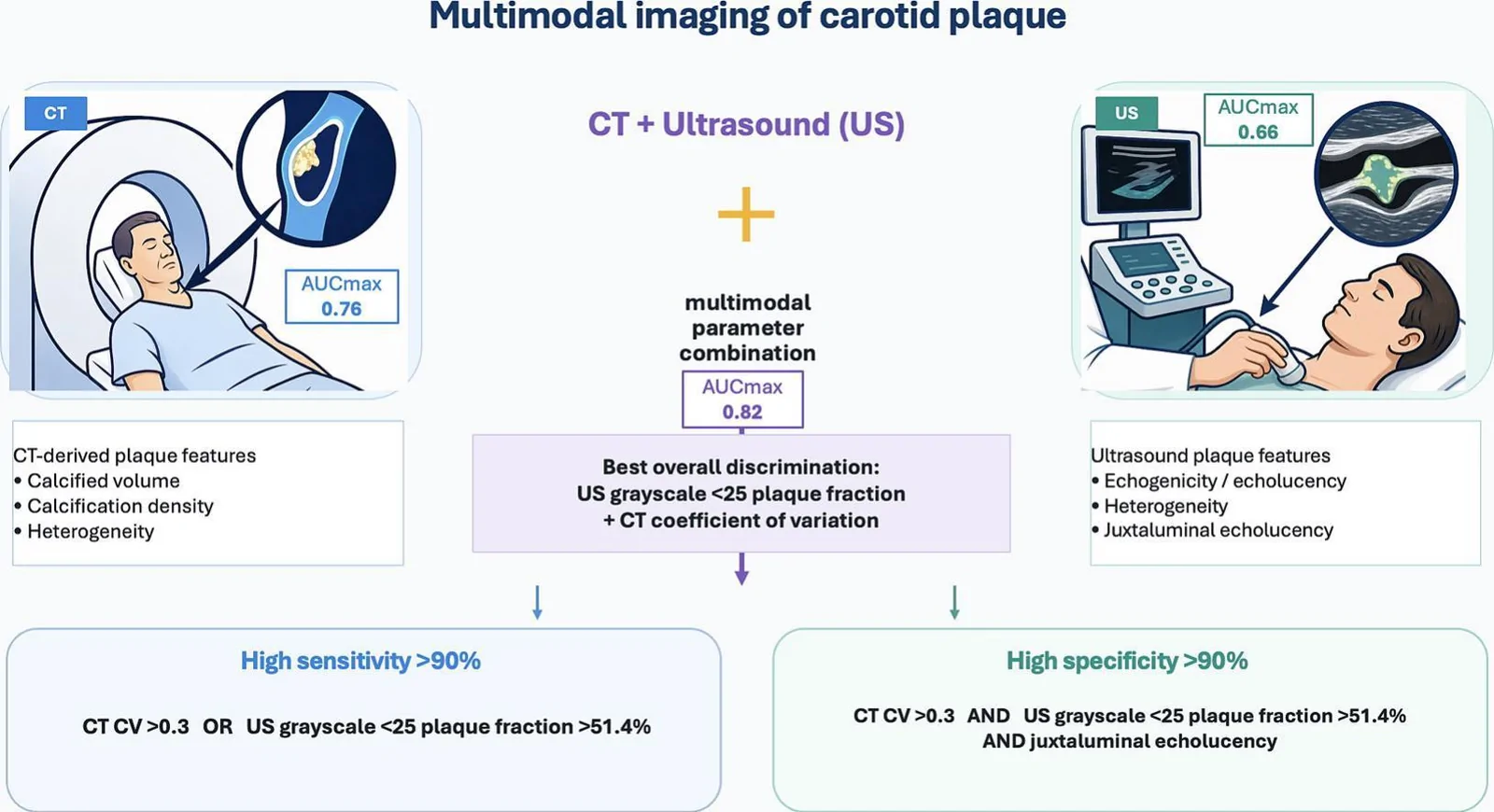

## 1 Introduction

Carotid stenosis causes a substantial proportion of ischemic strokes, and current decisions about carotid endarterectomy (CEA) are based mainly on stenosis severity and recent neurological symptoms [1], [2], [3], [4]. The benefit of CEA depends on accurate risk stratification, for instance using plaque imaging with ultrasound (US), computed tomography (CT) or magnetic resonance imaging (MRI) [5], [6]. Stroke risk is influenced by histopathologically defined plaque vulnerability, however in the absence of histopathology, the value of imaging features is often assessed using prospective occurrence of stroke [6], [7], [8], [9]. In general, a vulnerable atherosclerotic plaque is characterized by instability that confers a high propensity for rapid progression and thromboembolism, thereby increasing the risk of ischemic events [10]. Moreover, imaging features of plaques differ between patients with symptomatic and asymptomatic carotid stenosis, also providing ways to generate hypotheses on imaging characteristics for risk stratification before CEA [6], [7], [8], [9].

International guidelines recommend US as first-line imaging for carotid stenosis, with CT angiography or magnetic resonance angiography used to corroborate stenosis severity and support revascularization planning [3], [4], [11]. Although these routinely available modalities can also characterize plaque morphology, decisions regarding CEA remain driven primarily by neurological symptoms and stenosis severity; selected plaque features may support risk stratification (i.e. juxtaluminal black area on US or intraplaque hemorrhage on MRI), but standardized integration of plaque characteristics into routine decision-making has not been established [3], [4], [6]. US is widely available and can identify plaques from symptomatic patients according to lowered echogenicity, quantified by gray-scale median (GSM) <24.4 to 74 gray scales (GS) [12], [13], [14], [15], [16], [17]. Moreover, lowered echogenicity adjacent to the lumen, discrete white areas (DWA, hemosiderin deposits) or ulcerations are also more present in symptomatic patients [18], [19], [20]. CT requires contrast agent and ionizing radiation but can identify symptomatic patients according to Hounsfield unit (HU) density and the type and content of calcification, whereas higher calcification burden itself appears to be more common in asymptomatic patients [21], [22], [23], [24], [25]. Heterogeneity of GS on US or HU on CT may also provide two distinct features of plaques from symptomatic patients [18], [25], [26]. MRI can identify symptomatic patients by non-calcified plaque features but is time-consuming and less available and is thus only used in selected cases, but not routinely before CEA [27], [28]. Prior studies combining US, CT, and MRI demonstrated enhanced detection of symptomatic patients, but evidence on plaque-specific combinations remains limited [29], [30], [31].

We thus aimed to determine whether US-derived plaque features were associated with symptomatic status and whether combining US- and CT-derived parameters improved discrimination between patients with symptomatic and asymptomatic carotid stenosis. We hypothesized that selected US features would be associated with symptomatic status and that multimodal combinations would provide incremental diagnostic information.

## 2 Patients and methods

This hypothesis-generating single-center prospective cohort was conducted at the University Heart Center Freiburg - Bad Krozingen from April 2012 to June 2022 and followed good clinical practice. The study was approved by the ethics committee of the Albert-Ludwigs-University Freiburg (ID 439/14). Patients undergoing unilateral CEA were consecutively enrolled and included after written informed consent. Adult patients were included when they had an indication for CEA and no bilateral carotid stenosis. Neither atrial fibrillation (AF) nor a specific presumed stroke etiology was an exclusion criterion. Patients were excluded from the analysis only when CT or US had not been performed at our center or when the available images were of insufficient quality for plaque analysis. The indication for CEA was determined by a neurovascular board (including a neurologist, vascular surgeon, angiologist, cardiologist, anesthesiologist, and neuroradiologist) especially designed to decide whether patients with extracranial carotid stenosis should receive best medical therapy, stenting or CEA. In general patients were considered for CEA, when the extracranial stenosis was >50% with neurological symptoms or > 70%, surgical removal was deemed possible (location near the carotid bifurcation and not too distal), and stenting was not considered superior [3], [32]. Patients were classified as symptomatic when they had experienced stroke, transient ischemic attack, or retinal ischemia ipsilateral to the carotid stenosis within 6 months before CEA and the neurovascular board considered the carotid lesion the most likely etiology. Patients whose neurological event was judged more likely to reflect cardioembolism, including AF, small-vessel disease, or another non-carotid cause were retained in the cohort if CEA remained indicated but were classified as asymptomatic with respect to the carotid stenosis. AF status did not automatically determine eligibility or symptom classification; the board assigned the most probable etiology by consensus using all available clinical, cardiac, and neurovascular information.

**Fig. 1:**
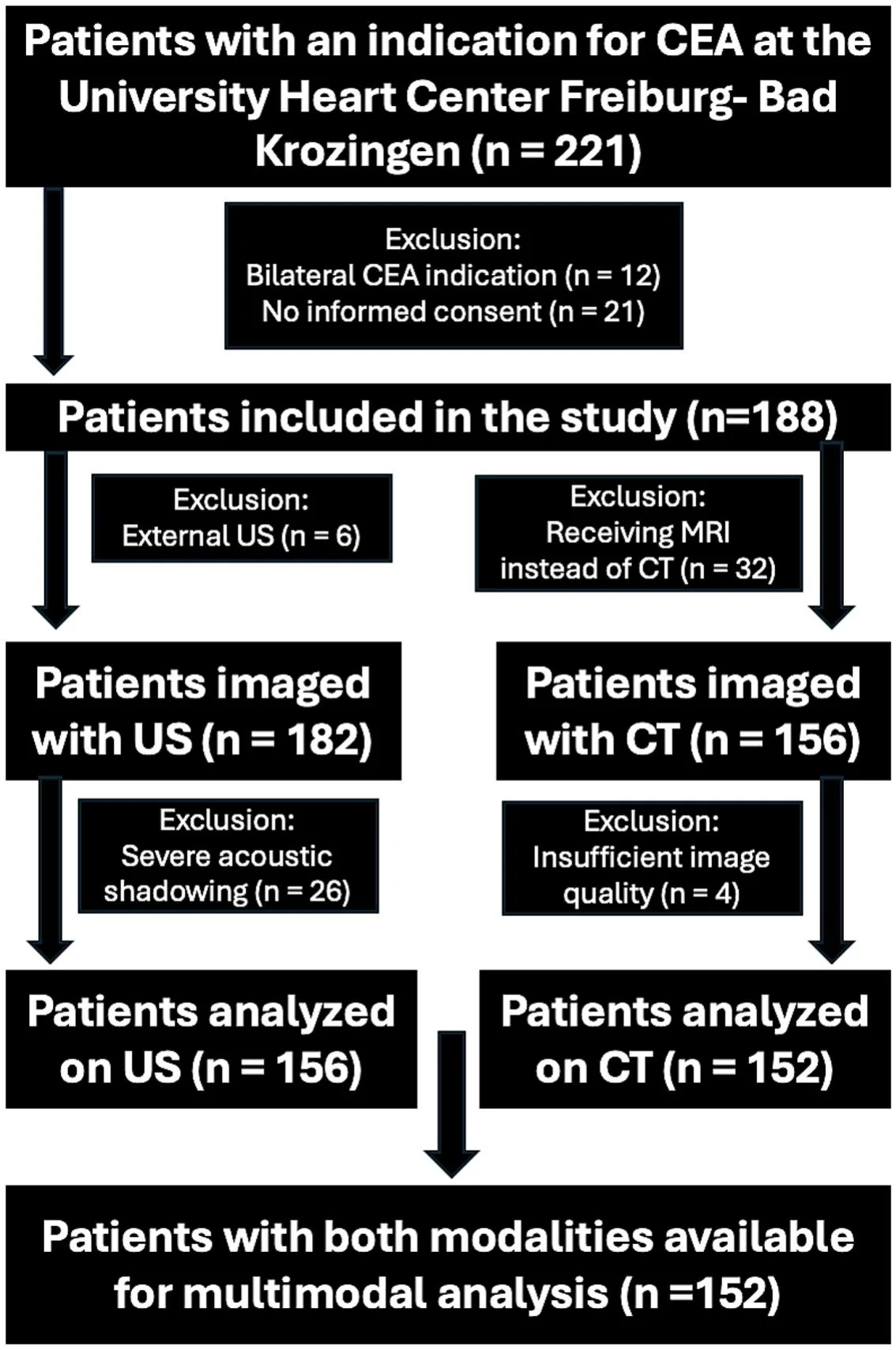
Flowchart. Flowchart of 188 patients who underwent carotid endarterectomy (CEA), showing availability and analyzability of ultrasound (US) and computed tomography (CT) data.

US evaluation of carotid stenosis was available in 182 patients (imaging performed at other institutions without access to data: *n* = 6). Twenty-six examinations were excluded due to severe acoustic shadowing, resulting in 156 patients for unimodal US analysis. CT was performed in 152 patients as previously described [25]. For multimodal analysis, 156 patients were included in total, of whom 152 underwent both US and CT (Fig. 1). Clinical characteristics and pre-operative neurological status were recorded from routine data.

### 2.1 US

US imaging was performed using a Philips IU22 system (L9–3 MHz transducer; PHILIPS, Hamburg, Germany) by an experienced ultrasonographer. Stenosis grading followed German Society for Ultrasound in Medicine (DEGUM) velocity criteria [33]. Patients were scanned on both sides of the neck and hemodynamically relevant stenoses were further examined (irrespective of the symptoms reported by the patient or previous US findings). B-mode settings were optimized for signal-to-noise ratio, and plaque images were acquired at the point of maximum luminal narrowing while avoiding acoustic shadowing and including the adventitial layer for later normalization of gray-scales. Images at the minimal lumen diameter were saved with and without color Doppler. When severe acoustic shadowing made adequate plaque assessment impossible, the patient was not further examined using US. Images were stored as JPEG files and analyzed in ImageJ (National Institutes of Health, Maryland, USA) by an experienced observer blinded to clinical symptoms, who was provided images of the plaques that were subsequently removed during CEA.

**Fig. 2:**
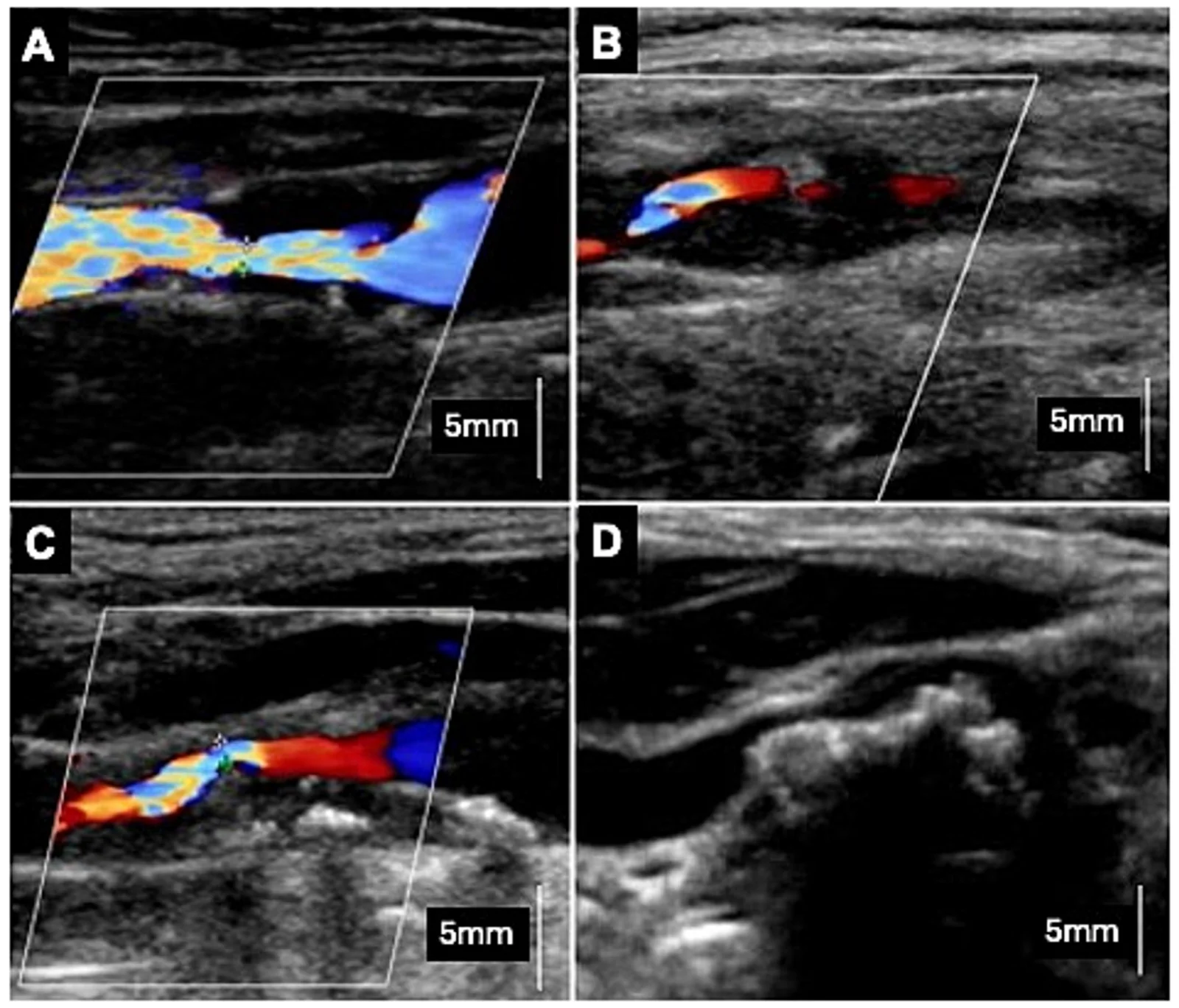
Carotid plaque echogenicity on ultrasound. Four patients with carotid stenosis comprised of plaques that are echolucent (A), more echolucent than echogenic (B), more echogenic than echolucent (C), and echogenic with acoustic shadowing (D) on ultrasound.

**Fig. 3:**
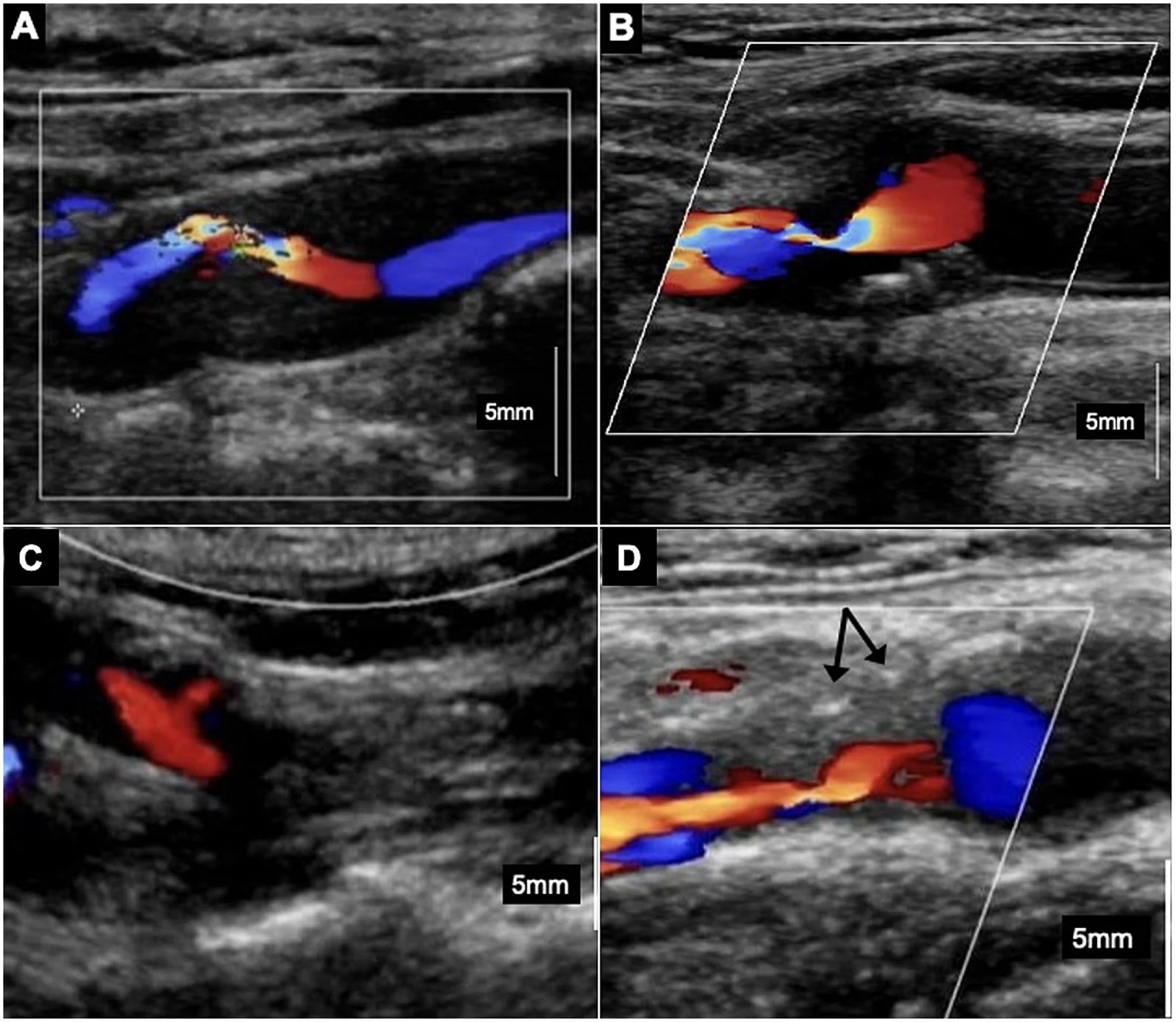
Carotid plaque juxtaluminal echolucency, Discrete White Areas and ulceration. A, B: Two patients with carotid plaques displaying juxtaluminal echolucency – i.e. echolucent plaque adjacent to the lumen on ultrasound. C: Patient with carotid stenosis by a plaque complicated with ulceration from the lumen into the plaque with doppler flow on ultrasound. D: Patient with a carotid plaque that contains two hyperechogenic Discrete White Areas (DWA) without acoustic shadowing on ultrasound.

Gray-scale (GS) values were normalized to the adventitial layer (reference = 190) [17]. A region of interest was placed around the plaque and GS histograms were generated. GS average, standard deviation, median (GSM), quartiles, and coefficient of variation (CV) were calculated (Fig. 2). Thus, US heterogeneity was evaluated quantitatively as the standard deviation divided by the mean GS value. Calcification with acoustic shadowing was graded on a subjective 0–3 score, however plaques with severe acoustic shadowing were already excluded. Additional plaque features - including echogenicity, juxtaluminal echolucency (significant echolucent plaque material adjacent to the vessel lumen, Fig. 3A; B), ulceration (evaluated where color Doppler images of the minimal lumen diameter were available, Fig. 3C), and DWA (small hyperechoic areas within the plaque without acoustic shadowing, Fig. 3D) - were assessed visually by a single observer [12], [19]. A gray-scale <25 was generally assumed echolucent based on the lower bound of the published literature [14]. No second independent US reading was available; therefore, interobserver agreement could not be estimated for qualitative US features.

### 2.2 CT

CT was performed as previously described in detail [25]. In brief, imaging was conducted on a 64-row multidetector CT scanner (SOMATOM Sensation Plus, Siemens, Germany) using 120 kV and 240 mAs with iomeprol (Imeron® 400; Bracco Imaging Deutschland GmbH, Konstanz, Germany), administered at 1 mL/kg body weight over 1.5 s. Images were reconstructed using a D26f Kernel, window and level were set to 400L/900W or 155L/50W and analyzed using J-Vision software (TIANI, Inc., Austria) by an experienced observer. Measurements included degree of stenosis, calcified plaque volume (defined as volume with >130 HU), HU-based density, heterogeneity expressed as CV (standard deviation divided by mean HU), and localization of calcification (deep/adventitial vs. superficial/luminal) [25]. Volumes were calculated using a cylindrical approximation.

### 2.3 Multimodal criteria combination

Candidate variables were selected from US features associated with symptomatic status in the present analysis and CT features identified in the prior volumetric analysis, according to their diagnostic accuracy [25]. The US candidates were the plaque proportion with GS <25 and juxtaluminal echolucency; the CT candidates were calcified plaque proportion and CT-derived CV. Calcification was included as an inversely associated discriminator, not as a positive risk marker. US-derived CV was also evaluated as a sonographic measure of heterogeneity but was inferior to CT and thus not included in combinations. For continuous (“metric”) combinations, plaque proportion with GS <25 and calcified plaque proportion were multiplied by 0.1, CT-derived CV was multiplied by 10, and parameters inversely associated with symptomatic status were directionally reversed before equally weighted averages were calculated. Receiver operating characteristic (ROC) analysis was used to identify score thresholds. Two-parameter combinations with higher areas under the curve (AUCs) were expanded to higher-order combinations.

For binary combinations, continuous variables were dichotomized at thresholds maximizing the Youden index in the present dataset: plaque proportion with GS <25 >51.4%, calcified plaque proportion on CT <66.0%, and CT-derived CV >0.3; juxtaluminal echolucency was inherently binary. Variables were combined using logical “AND” or “OR” rules. These thresholds were data-derived and were not externally validated. For the four participants without CT, multimodal combinations were calculated using US parameters only.

### 2.4 Statistics

All statistical analyses were performed using SPSS V25 (IBM, Armonk, NY, USA). The principal outcome was symptomatic status, as defined above. No formal sample-size calculation was performed for this exploratory study and the available consecutively enrolled prospective cohort was analyzed. Continuous variables are reported as mean ± standard deviation or point estimate with 95% confidence intervals; categorical variables are presented as absolute frequencies. Group differences were assessed with independent *t*-tests for continuous variables and χ^2^ tests for categorical variables. Two-sided *P* values ≤0.05 were considered statistically significant, but all analyses were interpreted as exploratory, since no adjustment for multiple testing was applied.

Variables showing group differences were entered into multivariable logistic regression models to estimate odds ratios (ORs) adjusted for age, continued smoking, coronary artery disease, chronic kidney disease, and stenosis degree. Diagnostic discrimination against the binary symptomatic-status reference standard was evaluated using AUC, sensitivity, and specificity. Thresholds were selected exploratorily to maximize the Youden index or to favor sensitivity or specificity, as stated for each analysis. AUCs were compared using DeLong's test. To assess potential confounding by cardioembolic events, an exploratory sensitivity analysis repeated the analyses after excluding all patients with AF (Supplementary Tables 1–3).

## 3 Results

### 3.1 Patient characteristics

Baseline characteristics for the 156 patients with analyzable US are shown in Table 1 (Table 1). AF was present in 22 patients: 9 of 56 symptomatic patients and 13 of 100 asymptomatic patients. New-onset AF was present in 3 patients, all in the asymptomatic group, and chronic AF in 19 patients (9 symptomatic and 10 asymptomatic). Symptomatic and asymptomatic patients showed no relevant differences in demographics or stenosis severity. Coronary artery disease was more prevalent among asymptomatic patients, reflecting the inclusion of patients who underwent CEA before coronary bypass surgery.

**Table 1:** Characteristics of patients receiving ultrasound.

Parameters	All *n* = 156	Symptomatic *n* = 56	Assymptomatic *n* = 100	*P*
	avg^a^ ± sd^b^ or n (%)	avg ± sd or n (%)	avg ± sd or n (%)	
NASCET stenosis (%)	68.2 ± 13.8	70.1 ± 12.9	66.2 ± 14.3	>0.100
Male gender	117 (75.0)	44 (78.6)	73 (73.0)	>0.100
Age (years)	70.1 ± 9.0	69.1 ± 11.3	71.0 ± 8.0	>0.100
Coronary artery disease	90 (57.7)	25 (45)	65 (65.0)	0.014
without bypass surgery patients	73 (48)	25 (44.6)	48 (48.0)	>0.100
Peripheral artery disease	50 (32.1)	13 (23.2)	37 (37.0)	0.081
Hypertension	147 (94.2)	52 (92.9)	95 (95.0)	>0.100
Hypercholesterolemia	147 (94.2)	52 (92.9)	95 (95.0)	>0.100
Diabetes mellitus II	41 (26.3)	13 (23.2)	28 (28.0)	>0.100
Smoking	42 (26.9)	17 (30.4)	25 (25.0)	>0.100
Renal insufficiency	33 (21.2)	13 (23.2)	20 (20.0)	>0.100
Atrial fibrillation	22 (14.1)	9 (16.1)	13 (13.0)	>0.100
of which new-onset	3 (1.9)	0 (0.0)	3 (3.0)	>0.100

a Average.

b Standard deviation.

### 3.2 Ultrasound associations with symptomatic status

**Fig. 4:**
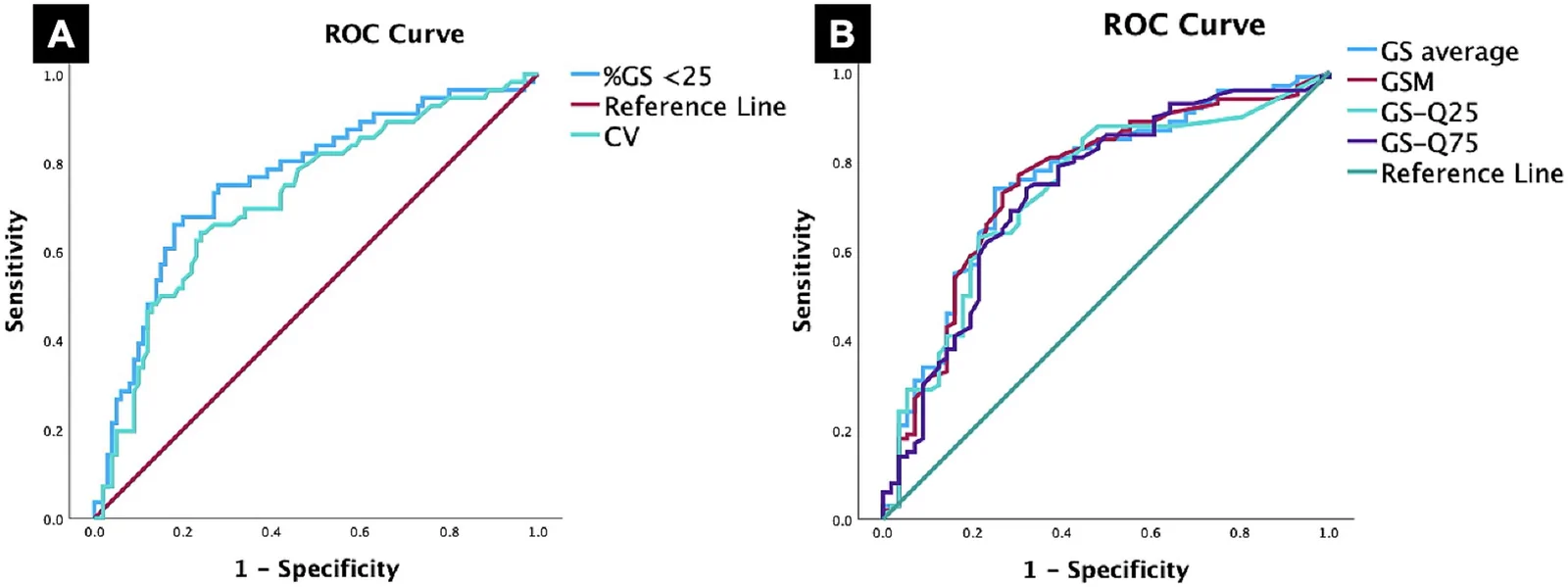
ROC-curve of carotid plaque grayscale echogenicity, echolucent proportion and heterogeneity on ultrasound. A: Receiver operating characteristic (ROC) curve of plaque echogenicity, assessed as the gray scale (GS) average, GS-median (GSM), 25th GS quantile (GS-Q25), and 75th GS quantile (GS-Q75) on ultrasound. B: ROC curve of echolucent plaque proportion assessed as the proportion with grayscale <25 (%GS <25) and heterogeneity, assessed as the coefficient of variation (CV, standard deviation divided by average) on ultrasound.

In adjusted logistic regression, lower GS-average (OR 0.70, 95% confidence interval 0.60–0.82), greater US heterogeneity (CV; OR 4.10, 2.00–8.30), and a larger plaque proportion with GS <25 (OR 1.40, 1.20–1.60) were associated with symptomatic status (Table 3). Qualitative features were also associated with symptomatic status: juxtaluminal echolucency (OR 9.90, 2.70–36.20), ulceration (OR 3.80, 1.30–10.20), and DWA (OR 7.40, 3.00–18.00). Descriptively, symptomatic patients had lower GSM (24.9 ± 27.1 vs. 48.0 ± 31.1 GS), lower GS-average (35.2 ± 25.3 vs. 57.9 ± 28.3 GS), and a greater plaque proportion with GS <25 (61.6 ± 35.9% vs. 30.9 ± 27.4%; all *P* < 0.001) (Table 2; Fig. 4A). US-derived CV was greater in symptomatic patients (1.20 ± 0.54 vs. 0.82 ± 0.48, *P* < 0.001). Juxtaluminal echolucency (23.2% vs. 3.0%, *P* < 0.001), ulceration (21.4% vs. 7.0%, *P* = 0.011), and DWA (37.5% vs. 8.0%, *P* < 0.001) were more frequent in symptomatic patients, whereas calcification with acoustic shadowing was less pronounced (score 1.0 ± 1.0 vs. 1.4 ± 1.1, *P* = 0.034).

**Table 2:** Plaque parameters on ultrasound of patients before carotid endarterectomy.

Parameter	All (n = 156)	Symptomatic (n = 56)	Asymptomatic (n = 100)	*P*
Metric	AVG^a^ ± SD^b^	AVG ± SD	AVG ± SD	
Grayscale median (GS^c^)	39.7 ± 29.7	24.9 ± 27.1	48.0 ± 31.1	<0.001
25th grayscale quantile (GS)	22.3 ± 22.4	13.1 ± 20.3	28.2 ± 23.5	<0.001
75th grayscale quantile (GS)	67.3 ± 37.7	48.1 ± 35.1	78.1 ± 39.1	<0.001
Grayscale average (GS)	49.8 ± 27.2	35.2 ± 25.3	57.9 ± 28.3	<0.001
Plaque with GS < 25 (%)	41.9 ± 30.5	61.6 ± 35.9	30.9 ± 27.4	<0.001
Coefficient of variation	0.96 ± 0.50	1.20 ± 0.54	0.82 ± 0.48	<0.001
acoustic shadowing (0–3)	1.2 ± 1.0	1.0 ± 1.0	1.4 ± 1.1	0.034


Binary	n (%)	n (%)	n (%)	*P*
Echolucency	17 (10.9)	9 (16.1)	8 (8.0)	>0.100
More echolucent	29 (18.6)	19 (33.9)	10 (10.0)	<0.001
More echogenic	52 (33.3)	19 (33.9)	33 (33.0)	>0.100
echogenicity	59 (37.8)	9 (16.1)	50 (50.0)	<0.001
Juxtaluminal echolucency	16 (10.3)	13 (23.2)	3 (3.0)	<0.001
Ulceration	19 (12.2)	12 (21.4)	7 (7.0)	0.011
Discrete white areas	29 (18.6)	21 (37.5)	8 (8.0)	<0.001

a Average.

b Standard deviation.

c Grayscale.

**Table 3:** Plaque parameters on ultrasound associated with neurologic symptoms.

Metric parameters	OR^a^	AUC^b^	Cut^c^	Sensitivity	Specificity
Grayscale median (GS^d^)	0.72* (0.62–0.84)	0.75 (0.67–0.84)	25.5	69.6 (62.4–76.8)	78.2 (71.7–84.7)
25th grayscale quantile (GS)	0.67* (0.54–0.83)	0.73 (0.65–0.82)	16.5	78.6 (72.2–85.0)	63.3 (55.7–70.9)
75th grayscale quantile (GS)	0.79* (0.71–0.88)	0.74 (0.65–0.82)	55.5	67.9 (60.6–75.2)	74.0 (67.1–80.9)
Grayscale average (GS)	0.70* (0.60–0.82)	0.76 (0.68–0.84)	42.4	75.0 (68.1–81.8)	74.1 (67.2–81.0)
Plaque with GS < 25 (%)	1.40* (1.20–1.60)	0.76 (0.68–0.84)	51.4	66.0 (58.6–73.4)	82.7 (76.8–88.6)
Coefficient of variation	4.10* (2.00–8.30)	0.72 (0.64–0.81)	1.0	64.2 (56.1–71.7)	76.6 (70.2–83.4)
Acoustic shadowing	0.70 (0.50–1.00)	0.61 (0.52–0.70)			


Binary parameters	OR	Sensitivity	Specificity
More echolucent	4.70 (2.10–8.80)	77.8 (70.7–83.6)	67.3 (59.6–74.2)
echogenic	0.20 (0.10–0.40)	88.9 (83.0–92.9)	46.2 (38.6–54.0)
Juxtaluminal echolucency	9.90 (2.70–36.20)	25.0 (18.9–32.3)	96.2 (91.9–98.3)
Ulceration	3.80 (1.30–10.20)	23.0 (17.1–30.2)	92.3 (87.0–95.5)
Discrete white areas	7.40 (3.00–18.00)	50.0 (42.2–57.8)	94.2 (89.4–96.9)

a Odds-Ratio.

b Area under the receiver operating curve.

c Threshold with maximum Youden-index.

d Gray scale.

* Odds-Ratio for an increase by 10.

**Table 4:** Plaque parameter associations with symptoms using combinatory ultrasound and computed tomography angiography.

Parameter combination averages	AUC^a^	Cut^b^	Sensitivity	Specificity
CVCT^c^; GS < 25^d^	0.83 (0.74–0.92)	4.0	80.0 (74.1–85.9)	81.7 (76.0–87.4)
CP^e^; GS < 25	0.76 (0.66–0.86)	4.2	83.3 (77.8–88.8)	64.7 (57.7–71.7)
CVCT; GS < 25; CP	0.81 (0.72–0.89)	3.7	86.1 (81.0–91.2)	69.1 (62.3–75.9)

Parameter combination binary				
CVCT; GS < 25	–	1	91.7 (87.6–95.8)	65.2 (58.2–72.2)
GS < 25; juxtaecho^f^	–	1	62.6 (52.6–76.2)	88.1 (83.3–92.9)
CVCT; GS < 25; juxtaecho	–	1	91.7 (87.6–95.8)	64.7 (57.7–71.1)

a Area under the receiver operating curve;

b Optimized with maximal Youden-Index;

c Coefficient of variation (CT) – binary threshold: >0.3.

d Proportion with grayscale <25 (US) – binary threshold: >51.4%.

e Calcified plaque proportion (CT).

f Juxtaluminal echolucency (US) – binary threshold: 1.

### 3.3 Ultrasound diagnostic discrimination

**Fig. 5:**
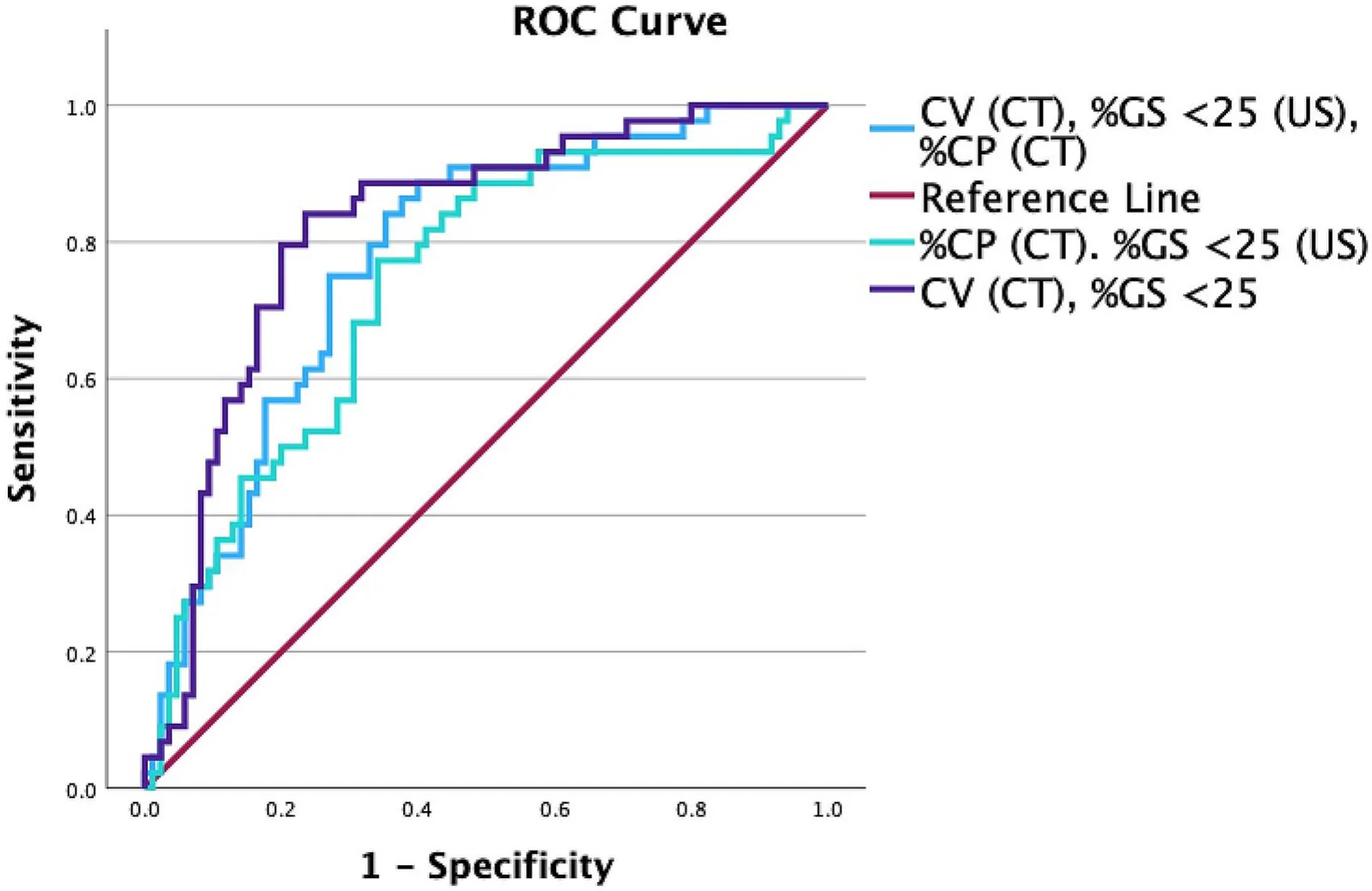
ROC-curve of carotid plaque multimodal imaging features. Receiver operating characteristic (ROC) curve of multimodal imaging parameters on computed tomography (CT) and ultrasound (US), assessed as weighted averages. Parameters were plaque heterogeneity on CT (coefficient of variation, CV), the proportion with grayscale <25 (%GS <25) on US, and the calcified plaque volume (CP) on CT.

Using symptomatic status as the reference standard, GS-average achieved an AUC of 0.76 (0.68–0.84) at a Youden-index threshold <42.4 GS, with 75.0% sensitivity (68.1–81.8%) and 74.1% specificity (67.2–81.0%). US-derived CV achieved an AUC of 0.72 (0.64–0.81) at >1.0, with 64.2% sensitivity (56.1–71.7%) and 76.6% specificity (70.2–83.4%). Plaque proportion with GS <25 achieved an AUC of 0.76 (0.68–0.84) at >51.4%, with 66.0% sensitivity (58.6–73.4%) and 82.7% specificity (76.8–88.6%) (Fig. 4B; Fig. 5). Juxtaluminal echolucency, ulceration, and DWA had specificities of 96.2%, 92.3%, and 94.2%, respectively, but lower sensitivities. A sensitivity-oriented threshold of plaque proportion with GS <25 >16.4% yielded 91.0% sensitivity and 47.6% specificity, whereas >71.0% yielded 45.7% sensitivity and 90.4% specificity. These thresholds were derived and evaluated in the same cohort.

### 3.4 Multimodal combination of ultrasound and computed tomography

Multimodal analyses included 156 patients, of whom 152 had both US and CT; the four participants without CT were handled as described in the Methods. The best unimodal US criterion was plaque proportion with GS <25 (AUC 0.76, 0.68–0.84).

The highest AUC was obtained by combining US-derived echolucent proportion with CT-derived volumetric heterogeneity (AUC 0.83, 0.74–0.92; threshold >4.0; sensitivity 80.0%, 74.1–85.9%; specificity 81.7%, 76.0–87.4%). The AUC was numerically higher than that of the best unimodal US criterion, but the difference did not reach statistical significance (0.83 vs. 0.76; *P* = 0.062). Adding calcified plaque proportion as a third parameter did not improve the AUC (0.81, 0.72–0.89; sensitivity 86.1%, specificity 69.1%) (Table 4; Fig. 5).

Other thresholds optimized for sensitivity or specificity are presented in supplemental Table 4. CT-derived CV >0.3 OR US-derived plaque proportion with GS <25 >51.4% yielded 91.7% sensitivity (87.6–95.8%) and 65.2% specificity (58.2–72.2%). Requiring CT-derived CV >0.3 AND plaque proportion with GS <25 >51.4% AND juxtaluminal echolucency yielded 94.1% specificity (90.6–97.6%) and 36.1% sensitivity (29.0–43.2%).

## 4 Discussion

Several US-derived plaque features were associated with symptomatic status, and a simple combination of US-derived echolucent proportion and CT-derived volumetric heterogeneity yielded a higher AUC than the best US measure. Because symptomatic status—not histology or prospective stroke occurrence—was the reference standard, these findings should be interpreted as exploratory evidence supporting external validation.

### 4.1 Ultrasound

GS-average, plaque proportion with GS <25, and US-derived CV provided moderate discrimination, whereas juxtaluminal echolucency, ulceration, and DWA were highly specific but insensitive. This pattern is consistent with systematic reviews showing that US performance depends on the plaque feature and reference standard [27], [28], [34].

Lower echogenicity may correspond to lipid-rich or necrotic plaque components, and juxtaluminal echolucency may indicate vulnerable material close to the lumen [19], [35], [36]. The proportion of plaque with GS <25, showed a strong gradient: each 10% increase corresponded to a 40% higher likelihood of being symptomatic, likely due to larger lipid-rich necrotic cores with thin fibrous caps prone to rupture [36]. However, without histological reference, these results remain inferential. Heterogeneity was evaluated on US as CV and was associated with symptomatic status (OR 4.10; AUC 0.72). Although heterogeneity has already been linked to vulnerability, this study applied a simple quantitative and more objective method in patients [18], [37]. US-derived and CT-derived heterogeneity are related but not interchangeable, since echogenicity and CT attenuation are different physical properties and US was only assessed two-dimensionally. CT may therefore contribute spatial and compositional information not represented by the US measure.

Qualitative US features appear to be highly specific for symptomatic patients, but the absence of an independent second reading prevents assessment of reproducibility. DWA must also be distinguished from calcification by the absence of acoustic shadowing because the two findings may have opposing associations with symptomatic status. Moreover, contrast-enhanced US can further assess plaque neovascularization and enhancement, although a recent meta-analysis reported only moderate discrimination and substantial methodological heterogeneity [38].

Nevertheless, US parameters may entail even more potential, when included into multimodal analyses with other imaging modalities, especially when the patient's symptoms and stroke etiology are more complex.

### 4.2 Multimodal combination of ultrasound and computes tomography

The combination of two simple plaque-specific measures from routinely available modalities is the main finding of this study. US characterizes echogenicity without radiation, whereas CT provides high-resolution assessment of calcification and attenuation heterogeneity. Meta-analyses comparing imaging with histology or ipsilateral ischemia support these complementary strengths, while also showing that accuracy varies by feature and reference standard [27], [28], [39]. While currently CT typically follows suspicious US findings, this approach may not be ideal for plaque imaging, as both modalities may independently classify a plaque as high-risk. Alternatively, to maintain the cost-effectiveness and non-existent radiation exposure of initial US, a very sensitive plaque parameter may be chosen to decide for or against CT imaging, additional to the degree of stenosis. The present findings do not support routine additional CT solely for plaque characterization in every patient with carotid stenosis. A multimodal assessment may be most relevant when CT is already clinically indicated or available, when US is equivocal or technically limited, when symptoms and stenosis severity are discordant, or when additional plaque information could contribute to multidisciplinary assessment.

Prior studies suggested potential benefit but were limited in size or used models incorporating parameters unrelated to plaque morphology [29], [30]. Another study incoporated a comparable approach on ex-vivo specimens with similar results, but the correlation between ex-vivo and in-vivo US and CT has been shown to be poor [31], [40]. Imaging-based scores illustrate possible future directions but should not be conflated with the present analysis. A US-derived carotid plaque score has been associated prospectively with stroke and cardiovascular events in a population cohort, and the MRI-based IMPROVE model combines intraplaque hemorrhage with clinical factors to estimate future ipsilateral stroke risk in symptomatic disease [41], [42]. These prognostic designs differ fundamentally from our cross-sectional comparison of symptomatic and asymptomatic patients.

The strongest metric combination paired US-derived echolucent proportion with CT-derived CV and achieved >80% sensitivity and specificity. The combination of a predominantly echolucent plaque with high heterogeneity may correspond to plaques with high lipid content (that influences echogenicity) and diffuse necrotic and/or inflammatory processes that primarily influence heterogeneity and may thus identify patients at high risk of stroke. Binary rules may be easier to interpret, yet their thresholds and performance require independent validation.

Whether multimodal plaque assessment improves treatment selection or clinical outcomes requires prospective studies to compare the incremental value over stenosis severity and clinical characteristics.

### 4.3 Limitations

This study exclusively analyzed patients undergoing preoperative evaluation for revascularization, which limits generalizability but represents the most appropriate cohort for optimizing risk stratification before CEA. Additionally, the analysis included asymptomatic patients scheduled for coronary bypass surgery, introducing the risk of selection bias. Its cross-sectional design precludes assessment of future stroke risk. All CEA patients were retained irrespective of presumed stroke etiology, and symptom attribution was based on neurovascular-board consensus. AF is an independent source of cardioembolism and therefore represents an important potential confounder: in patients with AF, it was not possible to determine with certainty whether an ipsilateral neurological event arose from local carotid plaque morphology or cardiac embolism. No histopathological examination of retrieved intracranial thrombi was performed to distinguish these mechanisms. The sensitivity analysis excluding all patients with AF was performed to mitigate this concern, but it does not eliminate etiologic uncertainty in the primary analysis. The two-dimensional analysis of US may also appear artificial, as clinicians typically consider the plaque in its entirety. Multimodal imaging is more resource-intensive and must be balanced against potential clinical benefit, although both CT and US are already routinely performed for stenosis assessment. Metric combinations using weighted averages remain challenging to implement because they require harmonized extraction of complex parameters and currently lack adequate commercial software support. No formal a priori sample-size calculation was performed; therefore, the exploratory analyses may be susceptible to false-negative findings (type II error) because of limited statistical power. Conversely, the large number of exploratory comparisons without adjustment for multiple testing increases the risk of false-positive findings (type I error). The precision of the multimodal AUC comparison is consequently uncertain and should be interpreted cautiously; overall, the results remain hypothesis-generating rather than conclusive. Deriving thresholds and evaluating their performance in the same dataset may overestimate diagnostic performance, thus external validation is necessary. The absence of a histological reference standard for plaque composition represents an additional limitation. Finally, some assessments are susceptible to inter-observer variability.

## 5 Conclusion

Several quantitative and qualitative US-derived plaque features were associated with symptomatic status in patients with carotid stenosis undergoing CEA. Combining US-derived echolucent proportion with CT-derived volumetric heterogeneity yielded higher discrimination of symptomatic versus asymptomatic patients than the best US criterion. These findings are exploratory and do not demonstrate histological plaque vulnerability, prediction of future stroke, or clinical benefit. External validation should assess reproducibility and whether selected multimodal strategies improve clinical decision-making.

## CRediT authorship contribution statement

**Simon Stemmler:** Writing – review & editing, Writing – original draft, Visualization, Project administration, Methodology, Investigation, Formal analysis, Data curation, Conceptualization. **Martin Soschynski:** Writing – review & editing, Formal analysis, Data curation. **Martin Czerny:** Writing – review & editing, Formal analysis, Data curation. **Matthias Siepe:** Writing – review & editing, Formal analysis, Data curation. **Thomas Zeller:** Writing – review & editing, Validation, Supervision, Formal analysis, Conceptualization. **Dirk Westermann:** Writing – review & editing, Validation, Supervision, Conceptualization. **Roland-Richard Macharzina:** Writing – review & editing, Writing – original draft, Visualization, Validation, Supervision, Funding acquisition, Data curation, Conceptualization.

## Ethical statement

Written Informed consent was given by every patient prior to inclusion. The study was approved by the ethics committee of the University of Freiburg (ID 439/14).

## Declaration of Generative AI and AI-assisted technologies in the writing process

During the preparation of this work, the authors used ChatGPT 4.0 to improve readability, imagery and language. The authors reviewed and edited the content as needed and take full responsibility for the content of the publication.

## Declaration of competing interest

The authors declare that they have no known competing financial interests or personal relationships that could have appeared to influence the work reported in this paper.

## Data Availability

The data underlying this article are summarized in the article – detailed data (patient by patient) are available upon specific request to the corresponding author.
