# Supplementary material for: Multimodal ultrasound and computed tomography imaging may improve the discrimination between symptomatic and asymptomatic patients with carotid stenosis
Source: Am Heart J Plus. 2026 Sep 16;70:100898. doi: 10.1016/j.ahjo.2026.100898 (PMC13595128; doi:10.1016/j.ahjo.2026.100898)
Supplement: Supplementary file 1 [file mmc1.docx]

**Supplemental Figure 1. Carotid Plaque with large proportion of grayscale <25**

Patient with carotid stenosis from plaque with a 90% proportion measuring below a grayscale of 25 on ultrasound.

**Supplementary Table S1. Plaque parameters on ultrasound of patients before carotid endarterectomy after exclusion of patients with atrial fibrillation**

| **Parameter** | **All (n=134)** | **Symptomatic (n=47)** | **Asymptomatic (n=87)** | **p** |
| --- | --- | --- | --- | --- |
| **Metric** | **AVGᵃ ± SDᵇ** | **AVG ± SD** | **AVG ± SD** |  |
| Grayscale median (GSᶜ) | 40.2 ± 31.5 | 25.7 ± 27.4 | 48.1 ± 30.9 | <0.001 |
| 25th grayscale quantile (GS) | 22.9 ± 23.2 | 13.5 ± 20.1 | 28.0 ± 23.3 | <0.001 |
| 75th grayscale quantile (GS) | 68.0 ± 40.3 | 48.8 ± 35.7 | 78.4 ± 39.0 | <0.001 |
| Grayscale average (GS) | 50.2 ± 29.1 | 35.7 ± 25.6 | 58.0 ± 28.0 | <0.001 |
| Plaque with GS<25 (%) | 41.2 ± 33.5 | 60.8 ± 35.4 | 30.6 ± 27.2 | <0.001 |
| Coefficient of variation | 0.94 ± 0.53 | 1.18 ± 0.55 | 0.81 ± 0.47 | <0.001 |
| Acoustic shadowing (0–3) | 1.2 ± 1.1 | 1.0 ± 1.0 | 1.4 ± 1.1 | 0.07 |
| **Binary** | **n (%)** | **n (%)** | **n (%)** | **p** |
| Echolucency | 15 (11) | 8 (17) | 7 (8) | 0.15 |
| More echolucent | 24 (18) | 16 (34) | 8 (9) | <0.001 |
| More echogenic | 45 (34) | 16 (34) | 29 (33) | 1.00 |
| Echogenicity | 50 (37) | 7 (15) | 43 (49) | <0.001 |
| Juxtaluminal echolucency | 14 (10) | 11 (23) | 3 (3) | <0.001 |
| Ulceration | 16 (12) | 10 (21) | 6 (7) | 0.02 |
| Discrete white areas | 24 (18) | 17 (36) | 7 (8) | <0.001 |

*ᵃ average; ᵇ standard deviation; ᶜ grayscale.*

**Supplementary Table S2. Plaque parameters on ultrasound associated with neurologic symptoms after exclusion of patients with atrial fibrillation**

| **Metric parameters** | **ORᵃ** | **AUCᵇ** | **Cutᶜ** | **Sensitivity, %** | **Specificity, %** |
| --- | --- | --- | --- | --- | --- |
| Grayscale median (GSᵈ) | 0.73 (0.62–0.86) | 0.74 (0.65–0.83) | 25.0 | 68.1 (53.8–79.6) | 78.2 (68.4–85.5) |
| 25th grayscale quantile (GS) | 0.68 (0.54–0.86) | 0.72 (0.63–0.81) | 16.0 | 76.6 (62.8–86.4) | 63.2 (52.7–72.6) |
| 75th grayscale quantile (GS) | 0.79 (0.70–0.89) | 0.73 (0.64–0.82) | 56.0 | 68.1 (53.8–79.6) | 73.6 (63.4–81.7) |
| Grayscale average (GS) | 0.71 (0.60–0.84) | 0.75 (0.66–0.84) | 42.0 | 74.5 (60.5–84.7) | 73.6 (63.4–81.7) |
| Plaque with GS<25 (%) | 1.4 (1.2–1.7) | 0.75 (0.66–0.84) | 50.8 | 66.0 (51.7–77.8) | 82.8 (73.5–89.3) |
| Coefficient of variation | 3.9* (1.8–8.4) | 0.71 (0.61–0.80) | 1.0 | 63.8 (49.5–76.0) | 75.9 (65.9–83.6) |
| Acoustic shadowing | 0.72 (0.50–1.04) | 0.60 (0.50–0.70) |  |  |  |
| **Binary parameters** | **OR** |  |  | **Sensitivity, %** | **Specificity, %** |
| More echolucent | 5.1 (2.0–13.1) |  |  | 76.6 (62.8–86.4) | 66.7 (56.2–75.7) |
| Echogenic | 0.18 (0.07–0.44) |  |  | 89.4 (77.4–95.4) | 46.0 (35.9–56.4) |
| Juxtaluminal echolucency | 8.6 (2.3–32.5) |  |  | 25.5 (15.3–39.5) | 96.6 (90.3–98.8) |
| Ulceration | 3.6 (1.2–10.8) |  |  | 23.4 (13.6–37.2) | 92.0 (84.3–96.0) |
| Discrete white areas | 6.5 (2.4–17.2) |  |  | 48.9 (35.3–62.8) | 94.3 (87.2–97.5) |

*ᵃ Odds ratio; ᵇ area under the receiver operating characteristic curve; ᶜ threshold with maximum Youden index; ᵈ grayscale.*

**Supplementary Table S3. Plaque parameter associations with symptoms using combinatory ultrasound and computed tomography angiography after exclusion of patients with atrial fibrillation**

| **Parameter combination averages** | **AUCᵃ** | **Cutᵇ** | **Sensitivity, %** | **Specificity, %** |
| --- | --- | --- | --- | --- |
| CVCTᶜ; GS<25ᵈ | 0.82 (0.72–0.91) | 4.1 | 78.7 (70.1–85.4) | 80.5 (72.4–86.6) |
| CPᵉ; GS<25 | 0.75 (0.64–0.85) | 4.0 | 81.6 (74.0–87.4) | 64.0 (54.8–72.3) |
| CVCT; GS<25; CP | 0.80 (0.70–0.89) | 3.8 | 84.2 (77.0–89.5) | 68.3 (59.3–76.1) |
| **Parameter combination binary** |  |  |  |  |
| CVCT; GS<25 | – | 1 | 89.5 (83.1–93.7) | 64.0 (54.8–72.3) |
| GS<25; juxtaechoᶠ | – | 1 | 61.4 (52.4–69.7) | 87.0 (79.6–92.0) |
| CVCT; GS<25; juxtaecho | – | 1 | 89.5 (83.1–93.7) | 63.4 (54.2–71.6) |

*ᵃ area under the receiver operating characteristic curve; ᵇ optimized with maximal Youden index; ᶜ coefficient of variation (CT) – binary threshold: >0.3; ᵈ proportion with grayscale <25 (US) – binary threshold: >51.4%; ᵉ calcified plaque proportion (CT); ᶠ juxtaluminal echolucency (US) – binary threshold: 1.*

**Supplemental Table S4. Sensitivity and specificity weighted plaque parameter associations with symptomatic status using combinatory ultrasound and computed tomography**

| **Metric parameter combination averages** | **Cut 1ᵃ** | **Sensitivity** | **Specificity** | **Cut 2ᵇ** | **Sensitivity** | **Specificity** |
| --- | --- | --- | --- | --- | --- | --- |
| CVCTᶜ; GS<25ᵈ | 2.8 | 91.4 (87.3–95.5) | 57.9 (50.6–65.2) | 4.9 | 48.6 (41.2–56.0) | 91.2 (87.0–95.4) |
| CPᵉ; GS<25 | 3.6 | 91.7 (87.6–95.8) | 51.5 (44.1–58.9) | 6.5 | 38.9 (31.7–46.1) | 91.2 (87.0–95.4) |
| CVCT; GS<25; CP | 3.0 | 91.7 (87.6–95.8) | 42.6 (35.3–49.9) | 5.5 | 36.1 (29.0–43.2) | 91.2 (87.0–95.4) |
| **Binary parameter combinations** |  |  |  |  |  |  |
| CVCT; GS<25 | 1 | 91.7 (87.6–95.8) | 65.2 (58.2–72.2) | – | – | – |
| CPᵉ; GS<25 | 1 | 91.7 (87.6–95.8) | 36.8 (29.7–43.9) | – | – | – |
| CVCT; GS<25; juxtaechoᶠ | – | – | – | 1 | 36.1 (29.0–43.2) | 94.1 (90.6–97.6) |

*ᵃ threshold optimized for sensitivity; ᵇ threshold optimized for specificity; ᶜ coefficient of variation (CT) – binary threshold: >0.3; ᵈ proportion with grayscale <25 (US) – binary threshold: >51.4%; ᵉ calcified plaque proportion (CT); binary threshold: <66.0%; ᶠ juxtaluminal echolucency (US) – binary threshold: 1.*
